# Expression of *Escherichia coli araE* and modified *lacY* genes in *Campylobacter jejuni* is not sufficient for arabinose transport

**DOI:** 10.1099/acmi.0.000042

**Published:** 2019-07-16

**Authors:** Amritha Ramesh, Naomi Ikeda, Sona Rubinchik, Andrey V. Karlyshev

**Affiliations:** ^1^ SEC Faculty, Kingston University, Kingston upon Thames, Penrhyn Road, KT1 2EE, UK

**Keywords:** *Campylobacter jejuni*, arabinose transport, inducible gene expression, LacY, AraE, green fluorescent protein

## Abstract

**Introduction:**

Unlike *Escherichia coli *, *Campylobacter jejuni* is unable to import a range of sugars, including arabinose, which makes common expression vectors, such as pBAD33, non-functional in these bacteria.

**Aim:**

The aim of this study was to investigate whether the *E. coli* transporters AraE and modified LacY (LacYA177C) would enable *C. jejuni* to uptake arabinose.

**Methodology and Results:**

The respective genes of *E. coli* were constitutively expressed in *C. jejuni* strain 11168H after integration into the chromosome via homologous recombination. Vectors carrying these genes also contained a reporter gene, *gfp*, under the control of the arabinose-inducible promoter, pBAD. These constructs were verified in *E. coli* by demonstrating the induction of *gfp* in the presence of arabinose. Integration of the genes into one of the rRNA gene clusters was verified by PCR and genome sequencing. The latter also confirmed that the inserted gene clusters contained no mutations. Expression of the *gfp* gene in the presence of arabinose inducer was monitored using fluorescence microscopy of colonies and fluorimetry using both whole cells and lysates.

**Conclusion:**

The results demonstrated the inability of *C. jejuni* to use arabinose transporters, which are fully functional in *E. coli *, suggesting a remarkable difference in the physiology of these bacteria.

## Introduction

*Campylobacter* is the leading cause of foodborne bacterial gastrointestinal diseases worldwide, including in the USA and the European Union [1]. It is prevalently found in the intestines of chickens, with this being the major source of transmission to humans; around 80 % of the reported human cases of campylobacteriosis are attributed to this means of infection [2]. Other transmission factors include consumption of contaminated water and contact with animals [3, 4]. The manifestations of campylobacteriosis, which is also regarded as an example of ‘travellers’ diarrhoea’, can range from mild to severe, depending on the strain and host susceptibility [5]. Typical symptoms include abdominal cramping, vomiting, headaches and diarrhoea, which can last up to a week. In rare cases post-infectional complications, known as Guillian–Barré syndrome or Miller–Fisher syndrome, can occur, where the neuromuscular system of a patient is affected, eventually leading to paralysis [6].

*Campylobacter* are small, Gram-negative and microaerophilic micro-organisms, which grow optimally at temperatures between 37 °C and 42 °C. They are predominantly spiral and rod-shaped organisms with a size range of 0.2–0.8 µm×0.5–5 µm [7]. Under limiting environment conditions, they tend to undergo a change in cell shape to become coccoid, which some researchers associate with a state of dormancy, and are regarded as being in a ‘viable but non-culturable (VBNC)’ state [8]. This state is characterized by the production of degradative enzymes, cell shrinkage and low metabolic activity [1, 9].

The molecular mechanisms of coccoid form formation in *C. jejuni* remain unknown. Some studies with other bacteria suggest a link between coccoid form formation and peptidoglycan (PG) biosynthesis [10–12]. In addition, coccoid form formation in a closely related bacterium, *Helicobacter pylori *, was found to be associated with expression of the gene *amiA*, which is involved in PG modification [13]. Due to the similarity of the biochemical, genetic and morphological properties of *C. jejuni* and *H. pylori *, it was suggested that the same gene may be also be responsible for the spiral–coccoid form transition in *Campylobacter* [14]. However, in contrast to *H. pylori *, which contains two copies of the *amiA* genes, there is only one copy in *C. jejuni *, making it impossible to construct and investigate the properties of a respective knockout mutant, as this gene appears to be essential. The problem could be alleviated by the construction of a conditionally lethal mutant, in which expression of this gene could be regulated. Therefore, the focus of this study was to develop an inducible gene expression system based on such a system, which is available for *Escherichia coli* and other bacteria, but not for *Campylobacter* [15–17]. In particular, the widely used pBAD system is not suitable for the latter because these bacteria are naturally refractory to arabinose uptake due to the lack of genes required for the transport of this sugar. In *E. coli *, arabinose is generally transported into the cells via specialized proteins such as AraE [18, 19]. Remarkably, it was also found that a modified lactose transporter, LacY (LacYA177C), was also able to transport arabinose, even in the absence of AraE [20]. In this study we explored the possibility of utilizing these two types of *E. coli* arabinose transporters for the construction of a derivative of *C. jejuni* that is capable of importing this sugar. These respective genes were integrated into an rRNA gene cluster of *Campylobacter* via homologous recombination by using the previously developed pRR system [21]. The genes were placed under the control of a constitutively expressed chloramphenicol resistance (*cam^r^*) promoter. Arabinose uptake was monitored by the expression of a *gfp* (green fluorescein) reporter gene placed under the control of pBAD [22, 23]. The constructs were verified via genome sequencing. The results of the study suggest that neither of the arabinose transport genes expressed in *C. jejuni* can give these bacteria the ability to translocate this sugar into the cytoplasm. This study focuses on investigating the possibility of developing a regulated gene expression system in *Campylobacter* via the introduction of the *E. coli* genes required for arabinose uptake. Since no such work with *Campylobacter* has been conducted, the study is important as it paves the way to better understand the biology of this pathogen and will assist in the development of novel molecular biology tools and antibacterial drugs.

## Methods

### Bacterial strains and growth conditions

The 11168H strain used in this study is a hypermotile derivative of strain NCTC 11168 [24, 25]. The strains were stored at −80 °C in Mueller–Hinton (MH) broth (Fluka) supplemented with 15 % glycerol. They were recovered by incubation at 37 °C under microaerophilic conditions in a controlled atmosphere incubator (Don Whitely) or in jars supplemented with a CampyGen gas generating kit (Oxoid) for 24 h. Strains were grown on Columbia blood agar (Oxoid) supplemented with 5 % defribinated horse blood and selective Skirrow supplement (Oxoid).

*E. coli* XL1 cells were used for molecular cloning, e.g. for the construction of pRRBCD-*egfp-lacY*A177C and pRRBCD-*egfp-araE* plasmids. *E. coli* NEB high-efficiency cells (C2566l) were used for transformation experiments. The strains were grown on Luria–Bertani (LB) agar (Oxoid) supplemented under aerobic conditions at 37 °C. Glycerol stocks of *E. coli* were made with LB broth (Oxoid) supplemented with 15 % glycerol and stored at −80 °C.

### Construction of recombinant plasmids

The integration vectors used in this study were derived from the pRR plasmid described elsewhere [21]^20^. They contained a reporter gene, *gfp*, under the control of the inducible arabinose promoter, pBAD, and *araE* and modified *lacY* genes constitutively expressed under the control of a chloramphenicol promoter.

The ClaI-SalI fragment of pBAD33 containing a regulatory region and an inducible promoter was cloned into the XbaI site of the pRR plasmid after blunt-ending with T4 DNA polymerase to produce pRRB. The orientation of the cloned fragment was verified by restriction digestion with EcoRV. The *cam^r^* gene cassette, isolated from pAV35 [26] by digestion with KpnI, was blunt-ended with T4 DNA polymerase and inserted into the blunt-ended XbaI site of pRRB to produce pRRBC. The orientation of the insert was determined by double digestion with SphI and ClaI. The pRRBC construct was also further verified by primer walking sequencing to ensure that there were no mutations. The XbaI fragment of pEGFP (BD Biosciences Clontech, Palo Alto, CA, USA) containing a *gfp* gene was blunt-ended and cloned into the blunt-ended KpnI site of the pRRBC to produce pRRBC-*egfp*. The correct orientation of this construct was determined by digestion with StyI.

**Fig. 1.**
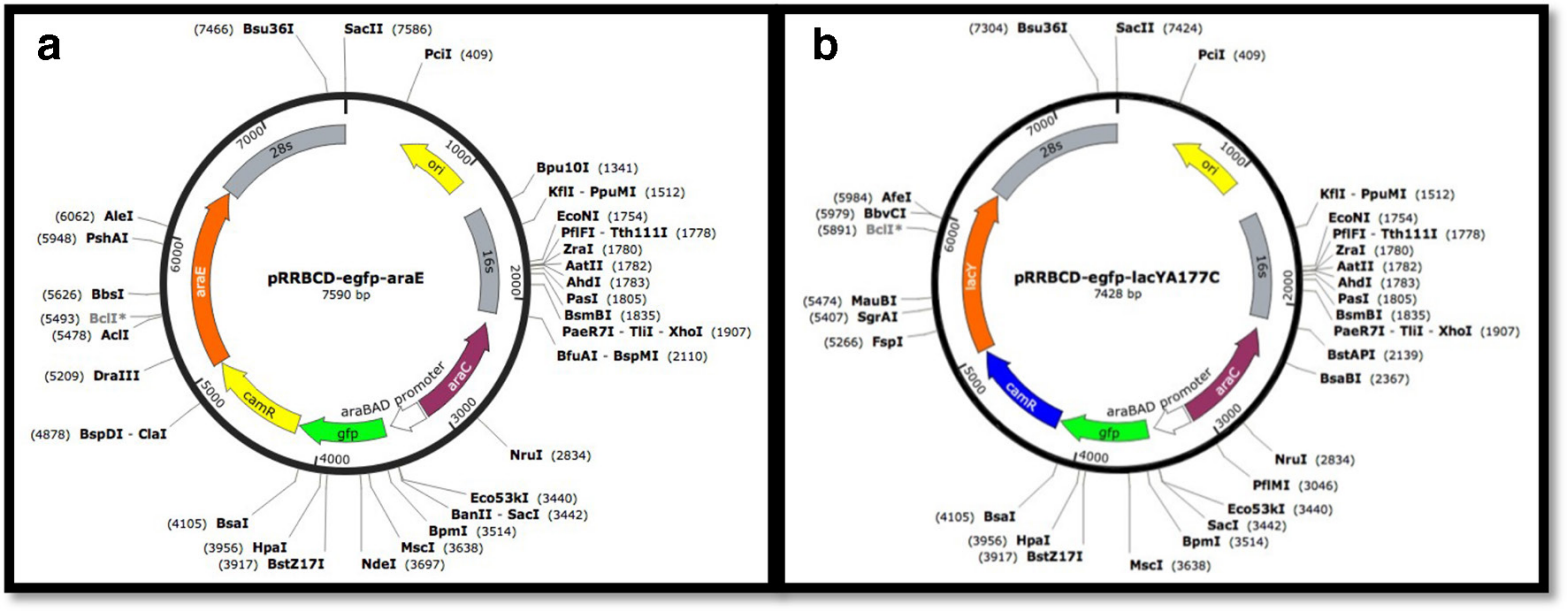
Maps of the integration vectors used in this study. (a) pRRBCD-*egfp-araE*. (b) pRRBCD-*egfp-lacY*A177C. The maps were created using Snapgene.

A DNA fragment with *E. coli* K12 *araE* gene (1.5 kb) was amplified using Phusion High-Fidelity DNA Polymerase and primers *araE*_for and *araE*_rev containing the SalI restriction site (Table 1). After cloning into pGEM-T vector and verification by sequencing, the SalI fragment with *araE* was cloned into the SalI site of pRRBCD-*egfp*. A derivative with correct orientation of the fragment was designated pRRBCD-*egfp-araE* (Fig. 1a).

**Table 1.** List of primers used in this study

Primer name	Sequence
ak233	GCAAGAGTTTTGCTTATGTTAGCAC
ak234	GAAATGGGCAGAGTGTATTCTCCG
ak235	GTGCGGATAATGTTGTTTCTG
ak237	TCCTGAACTCTTCATGTCGATTG
*lacY*_for	GTCGACAAGGAAATCCATTATGTACTATTTAAAAAAC
*lacY*_rev	GTCGACTTAAGCGACTTCATTCACCTGACGACGCAG
*cj1051*_for	AGTGTGTTAATTTCAAAACTCATAGCTAATAATC
*cj1051*_rev	GTAATTTTCTCTCCTAAGAATTCTTTCATAGC
*lacY*A177C_for	TGTCTCATCCTCGCCGTTTTACTC
*lacY*A177C_rev	ACAGCCAGAGCCCAGCCAGAAAAC
*araE*_for	GTCGAC AGGAGGAAAAA ATGGTTACTATC
*araE*_rev	GTCGACTCAGACGCCGATATTTCTCAAC

A 1.3 kb DNA fragment with the *E. coli* K12 *lacY* gene was PCR-amplified with primers *lacY*_for and *lacY*_rev (Table 1) and cloned into pGEM-T Easy vector to produce pGEM-T-*lacY*. The latter was then used as a template for another PCR using High-Fidelity DNA polymerase with primers *lacY*A177C_for and *lacY*A177C_rev primers (Table 1) to change the codon GCA for TGT, leading to a replacement of alanine at position 177 for cysteine. The resulting construct was verified by sequencing and designated pGEM-T-*lacY*A177C. A SalI fragment with gene *lacY*A177C was cloned into the SalI site of pRRBCD-*egfp* in correct orientation (verified by restriction analysis), producing pRRBCD-*egfp-lacY*A177C (Fig. 1b).

### Introduction of the gene cassettes into the chromosome via allelic replacements

The constructs were integrated into the chromosome of *C. jejuni* strain 11168H by using electroporation followed by plating of the transformation mixture onto selective medium as previously described [25]. The colonies were selected on either Cam (10 µg ml^−1^) or Tet (10 µg ml^−1^) plates, depending on the antibiotic resistance marker present (Fig. 1). The construct pRRBCD-*egfp-lacY*A177C was introduced into *C. jejuni* 11168H. However, due to unsuccessful attempts to deliver pRRBCD-*egfp-araE* construct into strain 11168H, a derivative of this strain, 11168H/*cj1051*, in which a gene encoding a restriction endonuclease was inactivated via insertional mutagenesis, was used. Mutant 11168H/*cj1051* was constructed by PCR amplification of this gene from *C. jejuni* 11168H using *cj1051*_for and *cj1051*_rev primers (Table 1) and Green GoTaq polymerase (Promega), followed by cloning into pGEM-T Easy to produce pGEM-T/*cj1051*. Insertion of a 2.8 kb NheI-XbaI fragment of pRRT plasmid with a tetracycline resistance gene *tet^r^* into the XbaI site of the latter produced pGEM-T/*cj1051*/*tet^r^*, which was then used for insertional mutagenesis of *C. jejuni *, leading to the construction of strain 11168H/*cj1051*. Integration of the gene cassettes into an rRNA gene cluster was verified by using PCR using Green GoTaq polymerase (Promega) with primer pairs ak233/ak237, ak234/ak237 and ak235/ak237 as described previously [21].

### Genome sequencing of *C. jejuni* 11168H *araE* and *lacY*A177C derivatives

The transformants were also checked by genome sequencing. The genome sequencing libraries were constructed by using the NEBNext Fast DNA Fragmentation and Library Prep Set for Ion Torrent (New England Biolabs). The genome sequencing was conducted using IonTorrent PGM (Life Technologies), the OT2 Hi-Q View 400 template, Ion PGM Hi-Q View 400 Sequencing kits and 316v2 chip (Life Technologies). The reads were mapped onto the reference genome sequence of *C. jejuni* strain NCTC 11168 (1 641 481 nt, accession number GCA_000009085.1) using CLC Genomics Workbench software (version 7.5), and the consensus sequences were extracted. The gaps between the consensuses were closed by using the contigs generated by the Torrent server SPAdes *de novo* assembly plugin (version 5.0.0.0.0). This allowed the generation of contiguous sequences, which were further verified by read mapping. The sizes of the genomes of *C. jejuni* 11168H/cj1051/*araE* and 11168H/*lacY*A177C derivatives were found to be 1 648 502 nt (102× coverage) and 1 645 651 nt (152× coverage), respectively. The sequencing revealed no errors (such as point mutations or indels) in the regions inserted into the rRNA gene clusters, confirming the full functionality of the *gfp*, *araE* and *lacYA177C* genes, as well as the regulatory genes and regions required for expression. Insertional inactivation of the gene *cj1051* in the *araE* derivative was also confirmed. The genome sequences were deposited in the NCBI’s GenBank database under the accession numbers CP022559.1 (11168H/cj1051/*araE*) and CP022439.1 (11168H/*lacY*A177C).

### Comparison of growth rates of the *C. jejuni* derivatives

In order to compare growth rates, the strains were suspended in BHI broth with a starting optical density (OD) of 0.1 and incubated on a shaker at 250 r.p.m., with OD measurements being taken at required intervals. For induction experiments, arabinose was added to a final concentration of 0.2 % after 6 h and incubation was continued for a further 21 h.

### GFP expression studies

Expression of the *gfp* gene in the *E. coli* strains and *C. jejuni* derivatives was tested using a fluorescence microscope (Nikon 80i Eclipse) with bacterial cultures grown on solid media with and without arabinose.

For fluorimetry, 150 μl of *E. coli* suspensions in LB broth with an initial OD of 0.5 was incubated in the wells of a 96-well non-treated polystyrene microtitre plate (Corning) for 2 h in an aerobic incubator at 37 °C with shaking at 120 r.p.m., followed by the addition of arabinose 0.1 % and subsequent incubation for 2 h. Similarly, *C. jejuni* strains were suspended in BHI broth with an initial OD of 0.5 and incubated on a shaking platform at 250 r.p.m. under microaerobic conditions at 37 °C for 2 h, followed by the addition of arabinose to 0.2 % and additional incubation as shown in the Results section. After completion of the induction cycle, the cells were centrifuged at 4000 ** *g* for 30 min and resuspended in PBS, and fluorescence readings of the whole cells were recorded. Similarly, the fluorescence of the lysed cells was measured after the cells were resuspended in a lysis mixture containing EDTA (5 mM) and protease inhibitor cocktail (Sigma Aldrich, p8849) according to the manufacturer’s instructions. Fluorescence was measured using BMG Labtech FLUOstar at an excitation wavelength of 485 nm and an emission wavelength of 520 nm.

## Results

### Expression of green fluorescence protein in *E. coli* strains containing the recombinant plasmids is dependent on the presence of arabinose

**Fig. 2.**
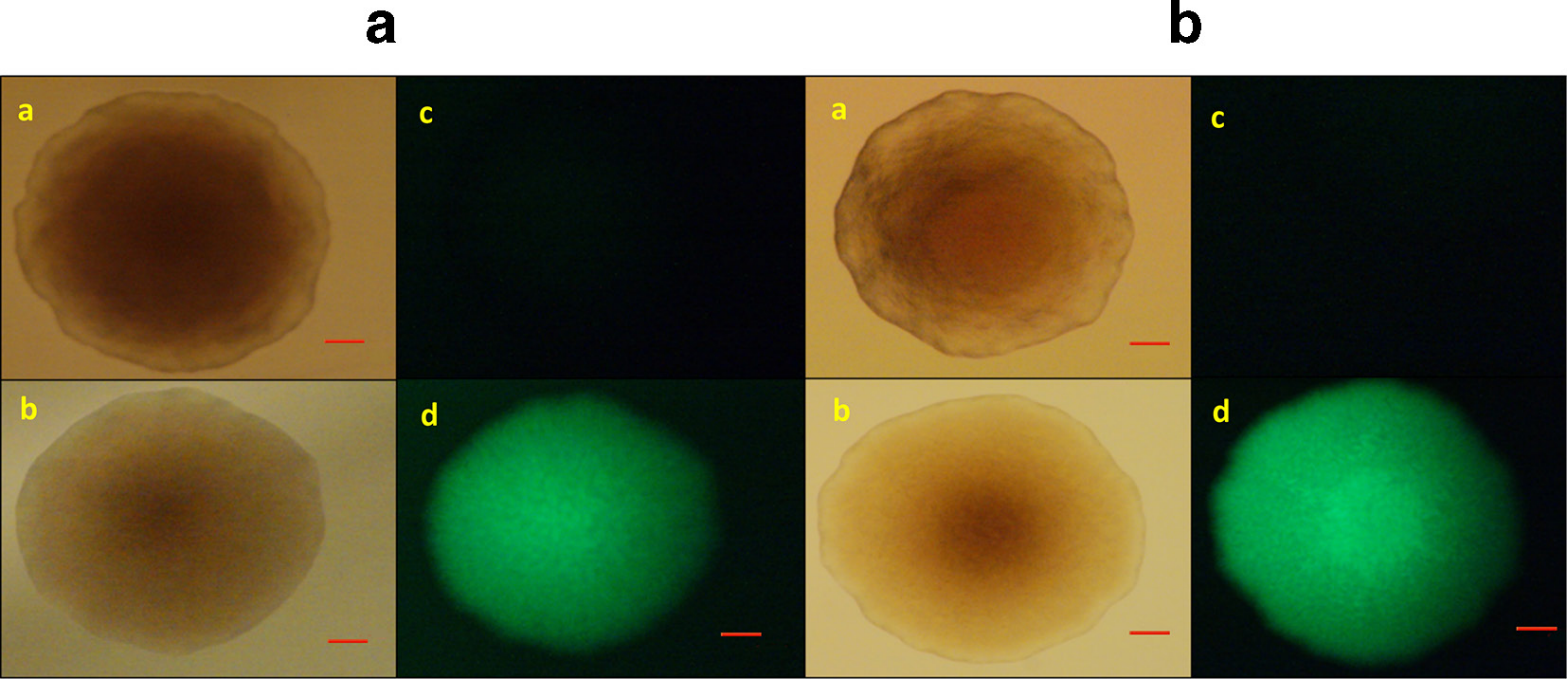
Fluorescence of *E. coli* strains in the presence of 0.1 % arabinose. (a) *E.coli*/pRRBCD-*egfp-araE*. (b) *E.coli*/pRRBCD-*egfp-lacY*A177C. a,c – without arabinose; b,d – with arabinose; a,b – under ambient light; c,d – fluorescence. Scale bar, 100 µm.

The fluorescence of *E. coli* strains carrying recombinant plasmids with *araE* and *lacY*A177C (Fig. 1) in the presence of 0.1 % arabinose was tested using a fluorescence microscope. The strains were grown on LB agar plates supplemented with chloramphenicol for 24 h. The colonies were found to be strongly fluorescent (Fig. 2).

**Fig. 3.**
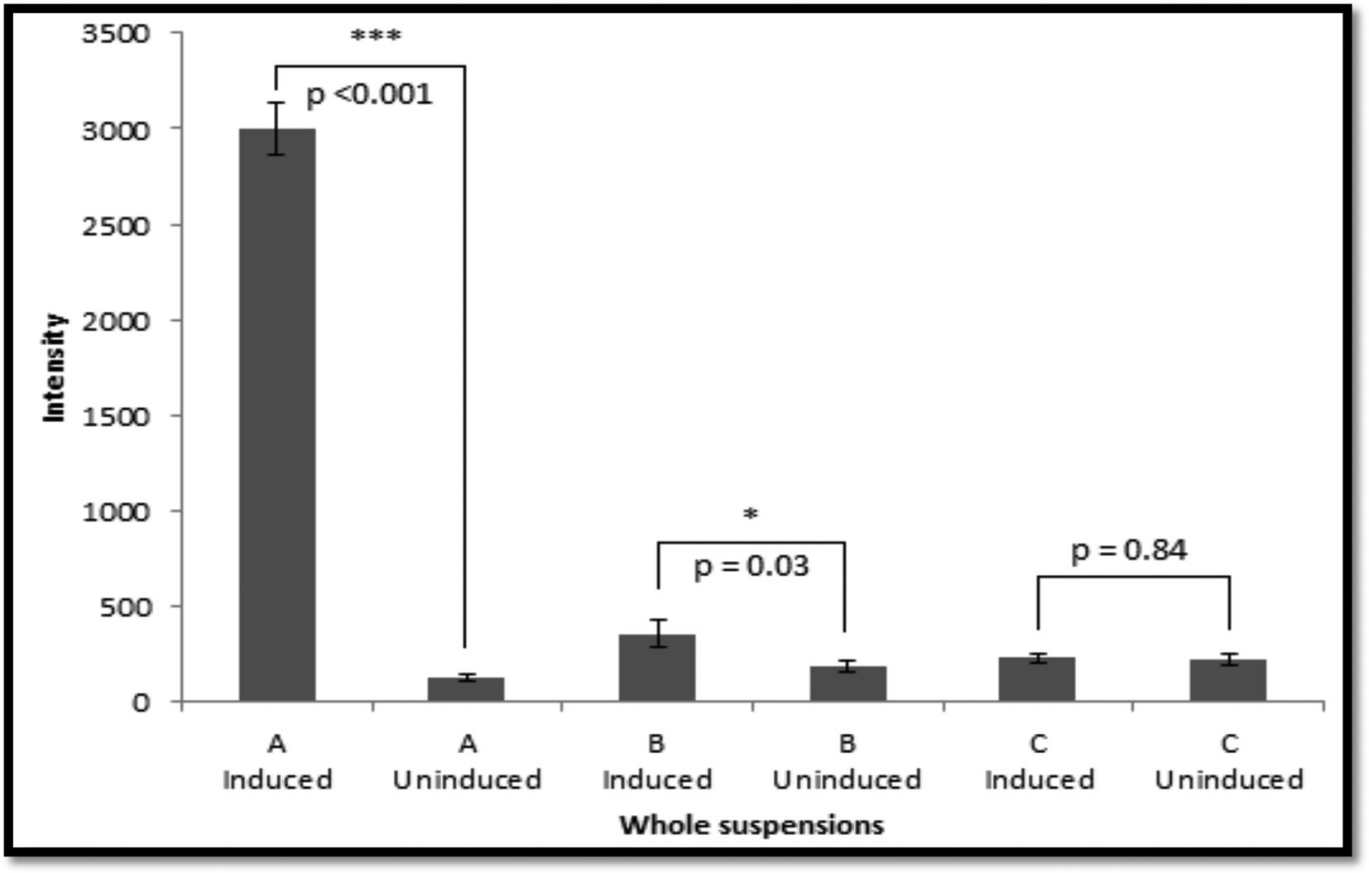
Fluorescence of whole bacterial samples in suspensions before and after induction. A, *E. coli */pRRBCD-*egfp-lacY*A177C; B, 11168H/*cj1051*/*egfp-araE*; C, 11168H/*egfp-lacY*A177C. * 0.01<*P*≤0.05, ** 0.001<*P*≤0.01, ***P≤0.001. Graph is a representation of three independent experiments comprising of three technical replicates. SEM values were used to represent error bars.

**Fig. 4.**
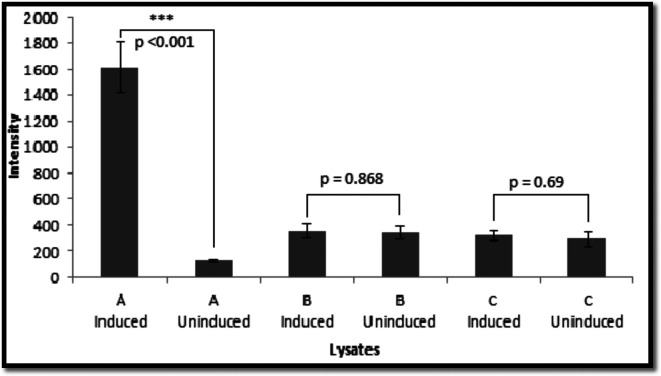
Fluorescence of bacterial samples in lysates before and after induction. (a) *E *. *coli */pRRBCD-*egfp-lacY*A177C. (b) 11168H//*cj1051*/*egfp-araE*. (c) 11168H/*egfp-lacY*A177C. *, 0.01<*P*≤0.05; **, 0.001<*P*≤0.01 ***, *P*≤0.001. The graph is a representation of three independent experiments comprising three technical replicates. SEM values were used to represent the error bars.

The expression of the *gfp* gene was also tested with these strains in liquid cultures using whole-cell suspensions and lysed cells. Induction by arabinose resulted in a significant increase in fluorescence compared with the uninduced control in both cases (Figs 3a and 4a).

### Confirmation of the presence of *araE* and mutant *lacY*A177C genes in the *C. jejuni* chromosome

**Fig. 5.**
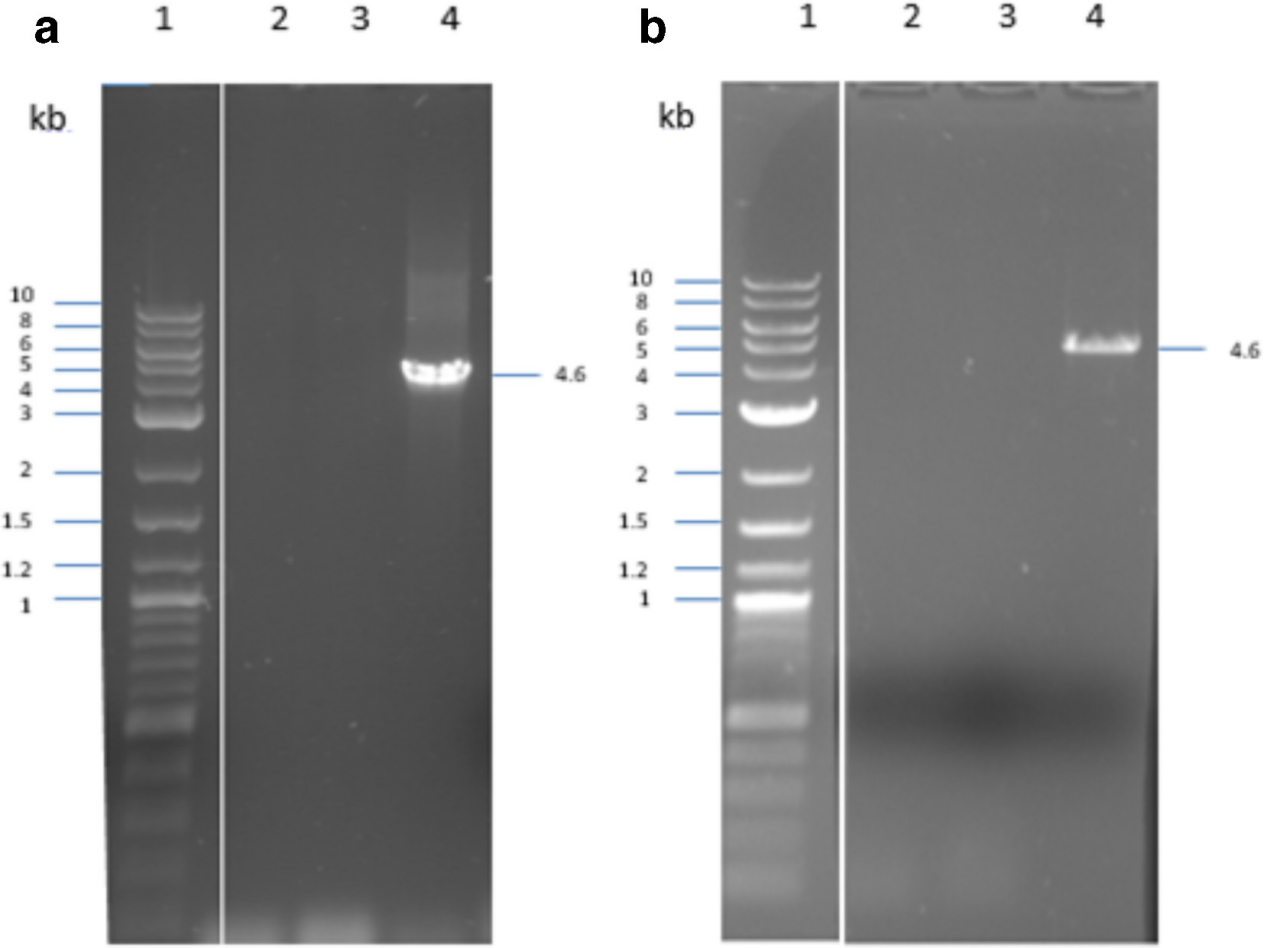
Verification of the insertion of gene cassettes carrying *araE* and *lacY*A177C genes into the *C. jejuni* chromosome by PCR using gel electrophoresis (1 % agarose). (a) 11168H/*egfp-lacY*A177C; (b) 11168H/*cj1051*/*egfp-araE*; 1, NEB 2-log ladder; 2, ak233/ak237; 3, ak234/ak237; 4, ak235/ak237.

**Fig. 6.**
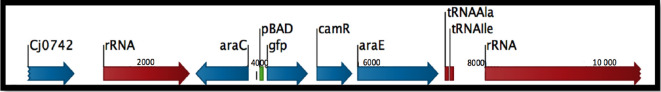
Representation of the chromosomal region between the 16S and 28S rRNA genes in the 11168H/*cj0151*/*egfp-araE* derivative.

**Fig. 7.**
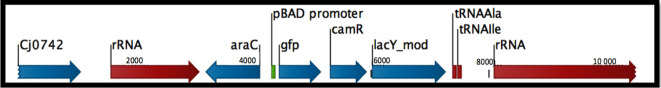
Representation of the chromosomal region between the 16S and 28S rRNA genes in the 11168H/*egfp-lacY*A177C derivative.

Due to the identity of the rRNA genes within the same cell, integration of the gene cassette into any of the three rRNA gene clusters could occur. PCR results (Fig. 5a and b) revealed that the integration of the cassette took place at the site close to the ak235 priming site in both cases, confirming that both cassettes were successfully inserted into the *C. jejuni* 11168H chromosome. The *C. jejuni* derivative strains were also subsequently verified by genome sequencing to ensure that there were no mutations. The images of the chromosomal regions of these *C. jejuni* derivatives are shown in Figs 6 and 7.

### Integration of the cassettes into the chromosome of *C. jejuni* has no effect on bacterial viability

**Fig. 8.**
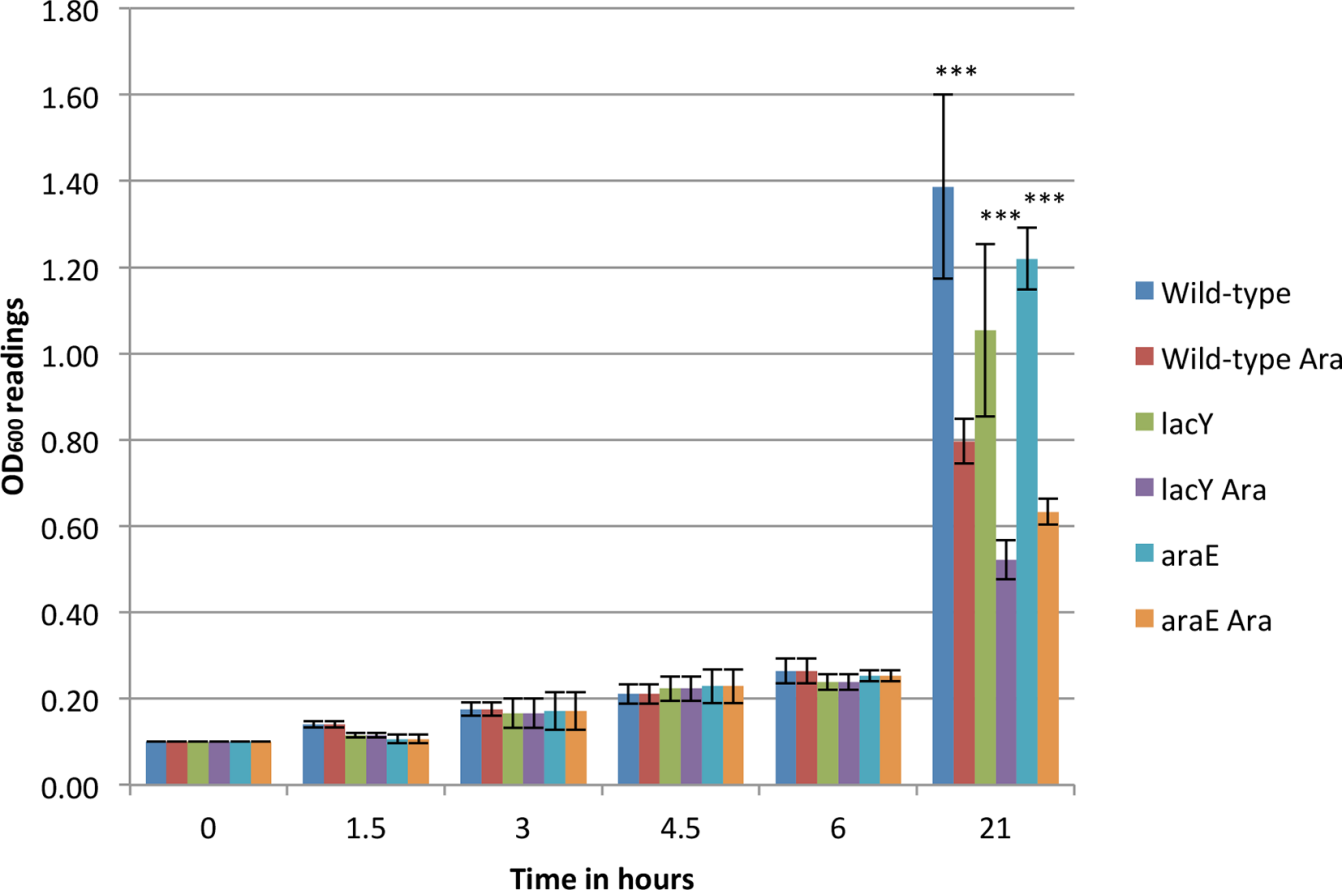
Comparison of growth rates of the 11168H strain of *C. jejuni* and its derivatives grown with and without arabinose. The graph is a representation of three independent experiments with three technical replicates. *, 0.01<*P*≤0.05; **, 0.001<*P*≤0.01; ***, *P*≤0.001. The *P* values to show the effect of arabinose on the growth rate of each strain when compared to uninduced cells are: 11168H, *P*<0.0001; 11168H/*egfp-lacY*A177C, *P*<0.0001; 11168H/*cj1051*/*egfp-araE*, *P*<0.0001. The *P* values to show the difference in the growth rate between the wild-type and derivative strains at the end of 21 h period are: 11168H and 11168H/*egfp-lacY*A177C, *P*=0.8; 11168H and 11168H/*cj1051*/*egfp-araE*, *P*=0.9.

The growth rates of the 11168H strain and its *araE* and *lacY*A177C derivatives in liquid culture, with and without arabinose, were compared by monitoring their OD_600_ values for 21 h. The was no difference between the samples for the first 6 h, whilst a statistically valid reduction in the growth rate was detectable after 21 h (Fig. 8). However, no difference was seen between the derivatives and the wild-type strain, suggesting that the insertion of the cassettes into the chromosome had no detrimental effect on bacterial viability.

### Addition of arabinose has no effect on expression of the *gfp* reporter in the derivative strains of *C. jejuni*

Since the colonies of the derivative strains of *C. jejuni* showed no detectable fluorescence when grown on arabinose-containing plates (data not shown), more accurate and sensitive analysis of fluorescence was conducted using a fluorimeter and liquid cultures. Since both derivatives of *E. coli* were behaving similarly and were producing strongly fluorescent colonies, only one positive control (* E. coli */*lacY*A177C) was used. Only a marginal difference was detected between the induced and uninduced samples when using whole-cell suspensions (Figs 3b and 4b), whilst no difference was observed for lysates (Figs 3c and 4c).

## Discussion

In this study, the pBAD promoter region of the pBAD33 vector was used in conjunction with a pRR plasmid system in the hope of constructing a strain that would allow regulated gene expression. The vectors used in this study were designed in such a way that the arabinose transporter genes (*araE* and *lacY*A177C) were independently (constitutively) expressed under the control of a chloramphenicol promoter and were carrying a reporter gene, *gfp*, under the control of an inducible pBAD promoter. Such a system was originally developed for exogenous gene delivery because commonly developed shuttle vectors could not be maintained in some strains of *C. jejuni* [21].

The vectors carrying these arabinose transporter genes were first tested and validated in *E. coli *. Due to the large size of the cassettes used for chromosomal integration, the efficiency of electroporation was quite low. In particular, transformation of pRRBCD-*egfp-araE* into *C. jejuni* could only be achieved after the introduction of a mutation into the gene *cj1051*, encoding a restriction endonuclease. Improved transformation efficiency after inactivation of this gene was reported previously [27]. However, in contrast to that earlier paper, which reported a 1000-fold increase in efficiency, when testing the derivative with a control plasmid pRRC only a marginal increase (2–5-fold) was detected. The reason for such a discrepancy remains unclear.

The results of this study revealed that, despite the expression of arabinose transporters, *Campylobacter* bacteria remained unable to import arabinose into the cytoplasm, suggesting a remarkable difference in the biology of these micro-organisms. This study suggests that there is a requirement for some other cellular structures or components for arabinose transport, which may be revealed in future studies.
